# The origin of life is a spatially localized stochastic transition

**DOI:** 10.1186/1745-6150-7-42

**Published:** 2012-11-24

**Authors:** Meng Wu, Paul G Higgs

**Affiliations:** 1 Origins Institute and Department of Physics and Astronomy, McMaster University, Hamilton, ON, L8S 4M1, Canada

## Abstract

**Background:**

Life depends on biopolymer sequences as catalysts and as genetic material. A key step in the Origin of Life is the emergence of an autocatalytic system of biopolymers. Here we study computational models that address the way a living autocatalytic system could have emerged from a non-living chemical system, as envisaged in the RNA World hypothesis.

**Results:**

We consider (i) a chemical reaction system describing RNA polymerization, and (ii) a simple model of catalytic replicators that we call the Two’s Company model. Both systems have two stable states: a non-living state, characterized by a slow spontaneous rate of RNA synthesis, and a living state, characterized by rapid autocatalytic RNA synthesis. The origin of life is a transition between these two stable states. The transition is driven by stochastic concentration fluctuations involving relatively small numbers of molecules in a localized region of space. These models are simulated on a two-dimensional lattice in which reactions occur locally on single sites and diffusion occurs by hopping of molecules to neighbouring sites.

**Conclusions:**

If diffusion is very rapid, the system is well-mixed. The transition to life becomes increasingly difficult as the lattice size is increased because the concentration fluctuations that drive the transition become relatively smaller when larger numbers of molecules are involved. In contrast, when diffusion occurs at a finite rate, concentration fluctuations are local. The transition to life occurs in one local region and then spreads across the rest of the surface. The transition becomes easier with larger lattice sizes because there are more independent regions in which it could occur. The key observations that apply to our models and to the real world are that the origin of life is a rare stochastic event that is localized in one region of space due to the limited rate of diffusion of the molecules involved and that the subsequent spread across the surface is deterministic. It is likely that the time required for the deterministic spread is much shorter than the waiting time for the origin, in which case life evolves only once on a planet, and then rapidly occupies the whole surface.

**Reviewers:**

Reviewed by Omer Markovitch (nominated by Doron Lancet), Claus Wilke, and Nobuto Takeuchi (nominated by Eugene Koonin).

## Background

When we consider the network of biochemical reactions that exists inside a living organism, it is important to realize that these reactions are in a dynamical steady state, rather than at thermodynamic equilibrium. The reaction system is maintained out of equilibrium because there is a continual input of energy and material (food) and because the rates of reactions are controlled by enzyme catalysts that permit desired reactions to occur much more rapidly than they would in a non-living mixture of molecules. The presence of biopolymer catalysts is an essential feature that distinguishes living systems from non-living chemical systems. Another key feature of living systems is that they are autocatalytic, *i.e.* they can reproduce and make more of their own components. Once again, it is biopolymers that allow organisms to do this. Cells use biopolymers to store the genetic information that allows them to produce the required catalysts for metabolism and to enable replication of the genetic polymers themselves. A commonly used definition of life is that it is a self-sustained system capable of undergoing Darwinian evolution [1]. As it is biopolymers that allow life to be self-sustained and enable heredity and evolution, we take the view that it is the emergence of autocatalytic biopolymer systems that is the key step in the origin of life. Thus, our aim is to understand how an autocatalytic biopolymer system could have emerged from a non-living chemical system.

It is usually thought that the interdependent system of DNA, RNA and proteins that sustains today’s cells is too complex to have arisen *de novo*. A prime candidate for a simpler biopolymer system that could have existed in early organisms is the RNA world hypothesis [2-5], which envisages that RNA sequences played both the genetic and catalytic roles. This is supported by the fact that RNA is the key component of the ribosome and by a large number of experimental studies of ribozymes *in vitro*[6-14]. In stronger forms of the RNA world hypothesis, it is argued that RNA sequences were the first autocatalytic biopolymers, and hence the first living system, rather than just an intermediate step between the origin of life and current life. It is then necessary to demonstrate that formation of functional RNAs was possible by non-living chemistry. Progress continues to be made on mechanisms of prebiotic synthesis of nucleotides and RNA oligomers [15-22]. In our view, the case that the origin of life occurred via an RNA world is quite strong, but it is still far from proven. Several authors have emphasized the difficulties associated with the RNA world and have considered alternative scenarios [23,24]. A review discussing why the RNA world is currently the best theory we have for the origin of life, despite acknowledged limitations, appeared very recently [25].

Although we will consider RNA world scenarios in this paper, the two most important points that we emphasize here are sufficiently general to apply if some other kind of replicating molecular system arose prior to the RNA world or instead of it. The first point is that the non-living and living states are two alternative dynamically stable states of the same chemical system. The second point is that the origin of life is a stochastic transition between these two states that is initiated by concentration fluctuations involving relatively small numbers of molecules in a localized spatial region.

We previously showed that two alternative dynamically stable states exist in a chemical reaction system that models RNA polymerization [26]. The model calculates the concentrations of monomers and polymers of different lengths. Monomer synthesis and polymerization reactions can occur at a small spontaneous rate in absence of ribozymes. Polymerization can also occur at a rapid rate due to catalysis by ribozymes, if these are present. In one stable state, which we call dead or non-living, the ribozyme concentration is negligible and the reactions proceed at their spontaneous rates only. In the other stable state, which we call living, there is a substantial ribozyme concentration and the polymerization rate is high, which causes autocatalytic synthesis of more ribozymes. We calculated the phase diagram as a function of the rate parameters for spontaneous and catalyzed reactions. We showed that there is a region where the reaction system is bistable (*i.e.* both solutions exist simultaneously) and also regions where only one or other of the two solutions is stable. We subsequently showed that a similar phase diagram exists for RNA systems with nucleotide synthase and polymerase ribozymes instead of polymerases [27] and in asymmetrical autocatalytic systems describing the origin of homochiral biopolymers [28]. In this paper, we will argue that the form of the phase diagram is a generic feature of models describing autocatalysis and the origin of life, and we will present a very straightforward model that shows this in its simplest and clearest form.

It is important that the real world must be in the bistable region of the phase diagram. The living state must exist in the real world, but it cannot be the only solution, because the origin of life is a difficult and rare event. We do not see spontaneously replicating systems popping up easily in every test tube or puddle. The existence of two stable states is also demonstrated by the fact that it is easy to kill an organism by depriving it of some required substance, but the organism is not easily brought back to life if the substance is resupplied (*e.g.* an organism that died of suffocation is not brought back to life by giving it oxygen). We presume that the world began in the non-living state, and that it was possible, but difficult, for a transition to occur to the living state. The question is how the transition could have occurred.

We previously argued that stochastic concentration fluctuations are essential for the origin of life [26]. In a large, well-mixed chemical system, we can write down deterministic ordinary differential equations to describe the concentrations of different types of molecule. The non-living system is dynamically stable for ever in such a deterministic case. In a system of finite volume, with finite numbers of molecules, stochastic fluctuations can occur that allow the system to jump from the non-living to the living state. In the RNA polymerization model, it is presumed that only fairly long RNA sequences have any chance of being functional ribozymes. Such sequences are very rare, and may only be present in a handful of copies, even though monomers and short oligomers may be common. Stochastic fluctuations in the ribozyme concentration are thus important, even if the monomer concentration is effectively deterministic.

In this paper, we want to investigate more carefully the factors that determine the time required for the stochastic transition to occur. We observed previously that the transition to life occurs most easily in systems of intermediate size [26]. If the system is too small, it is very difficult to create sequences long enough to be ribozymes. If the system is too large, the concentration fluctuations are too small to permit the transition to occur. It is clear, however, that there is something missing from this story. In reality, molecules have a finite diffusion rate. Molecules have a finite lifetime before they are broken down or destroyed. Only molecules relatively close to one another on the surface of a planet have any chance of interacting with one another. There must be a length scale controlled by diffusion above which different regions of space are effectively independent of one another on the time scale of the molecular lifetime. The assumption that the system is well mixed breaks down at larger lengths scales than this. Our aim in this paper is therefore to study two-dimensional spatial models (representing the surface of a planet) in which reactions occur locally and there is a finite rate of molecular diffusion. We will show that the concentration fluctuations that lead to the origin of life can occur locally in any one region of space. Once the stochastic transition has occurred in one place, the living state can then spread deterministically across the rest of the surface.

## Results

### A generic phase diagram for replicating molecules

In this section, we briefly summarize our RNA polymerization model [26] and show how the phase diagram is obtained in this case. We then introduce a very simple generic model for replicating molecules and show that the phase diagram is equivalent.

In the RNA system, we suppose that precursor “food” molecules are available in the environment at concentrations *F*_*1*_ and *F*_*2*_. We suppose that monomers, denoted by *A*, can be synthesized from *F*_1,_ with rate constant *s*. These monomers can react with *F*_*2*_ to produce activated monomers, A*, with rate constant *a*. RNA polymers of length *n* are denoted *A*_*n*_. An activated monomer can react with a polymer to extend its length by 1 with rate constant *r*. All molecules can escape from the system at a rate *u*. If the system is well-mixed, it can be described by the following ordinary differential equations:

(1) $$\frac{dA}{dt}=sF_{1}-aF_{2}A-rAA^{*}-uA$$

(2) $$\frac{dA_{n}}{dt}=rA^{*}\left(A_{n-1}-A_{n}\right)-uA_{n}$$

(3) $$\frac{dA^{*}}{dt}=aF_{2}A-rA^{*}\left(A+P\right)-uA^{*}$$

In Equation 3, *P* is the total polymer concentration of all lengths: $P=\sum_{n\geq 2}A_{n}$. We assume that there is a minimum length *m* above which polymers have the possibility to act as catalysts for the polymerization step. The concentration of polymers of at least length *m* is $P_{m}=\sum_{n\geq m}A_{n}$. The polymerization rate constant is

(4) $$r=r_{0}+kP_{m}\text{,}$$

where *r*_0_ is a small spontaneous polymerization rate and *kP*_*m*_ is the term due to ribozyme catalysts. The constant *k* is the catalytic efficiency of the long polymers, which may be written as *k = k*_*0*_*f*, where *f* is the fraction of the long polymers that function as catalysts and *k*_*0*_ is the catalytic efficiency of a functional catalyst.

**Figure 1:**
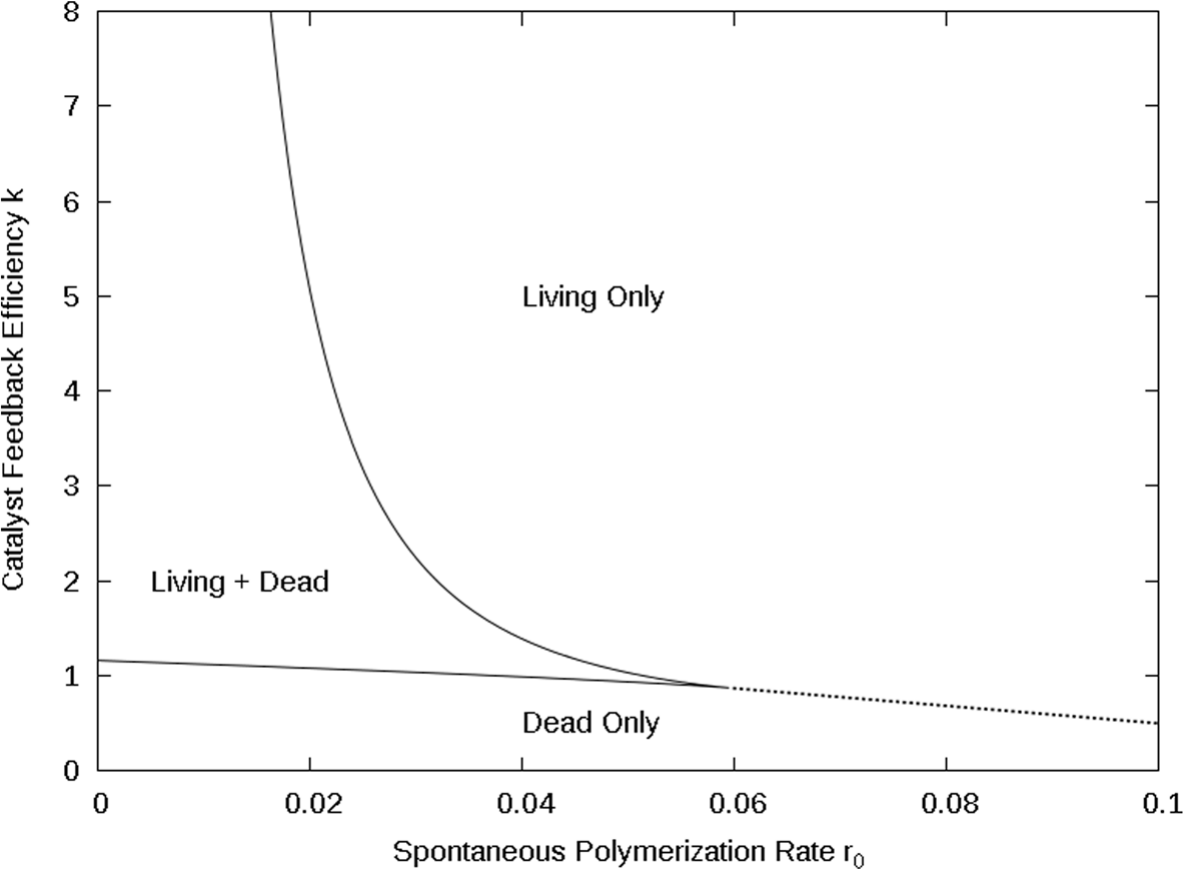
Phase diagram for the RNA polymerization model as function of catalyst feedback efficiency *k* and spontaneous polymerization rate *r*_0_ with the other parameter set as: *m*=5, *s*=10, *a*=10 and *u*=1.

We refer to Eqn. 4 as the feedback equation, because it says that the presence of catalysts feeds back into the polymerization rate and increases the rate of formation of more catalysts. The stationary states of the molecular concentrations can be obtained from Equations 14 using the methods described previously [24]. The key point is that there is a dead (or non-living) state in which *P*_*m*_ is very small and *r ≈ r*_*0*_, so that polymerization occurs at the spontaneous rate, and there is a living state in which *kP*_*m*_*>> r*_*0*_, so that polymerization is autocatalytic and occurs at a much higher rate than the spontaneous rate. Figure 1 shows a phase diagram as a function of *k* and *r*_*0.*_ We are most interested in the region where both states are stable. The bistable region occurs if *r*_*0*_ is fairly small and *k* is fairly large. If *k* is too small, only the dead state is stable, and if *k* is too large, only the living state is stable. Furthermore, if *r*_*0*_ is large then there is only one stable state for all values of *k*, and this changes smoothly from dead to living as *k* is increased across the dotted line in Figure 1. An example of this phase diagram for different parameter values has previously been given [26]. We have also considered the case where feedback is in the monomer synthesis rate [27] and have extended the model to deal with the possibility of chiral monomers [28]. Bistable regions were also found in these cases. A bistable region equivalent to ours was observed in related models for autocatalytic polymers [29-31], although no explicit phase diagram was drawn. When designing our original model, we were aware of the toy model for the origin of life by Dyson [32]. This model is described in a very abstract way with no explicit chemical reaction equations. Nevertheless, the central ideas of living and dead stable states and a stochastic transition between these states exist in this model, and the phase diagram of Dyson’s model also has a bistable region similar to ours.

Having seen that the kind of phase diagram in Figure 1 occurs in many different models, we now wish to introduce the simplest model we can that has the same phase diagram. The simplified model will then be useful to investigate the dynamic properties of the stochastic transition to life in the following sections of this paper.

We consider a single type of replicating molecule whose concentration is *ϕ*. Replicators can appear at rate *s*, representing a slow rate of spontaneous synthesis by random polymerization. This process is independent of the replicator concentration. Existing replicators may be copied with a rate constant *r*, representing a process of non-living template-directed synthesis. This process is proportional to the current replication concentration, process is proportional to the current replication *ϕ*. Replicators may also act as polymerases that catalyze replication by using another replicator as a template. The rate constant for this catalytic process is *k* and this process is proportional to the square of the current concentration, *ϕ*^2^. The increase in replicator concentration is limited by finite resources. The simplest way to model this is to assume a finite carrying capacity of the system, corresponding to a concentration *ϕ* = 1, and to multiply all the growth rates by a factor (1-*ϕ*). Finally, replicators die (or are destroyed or removed from the system) at a rate *u*. The deterministic dynamical equation for *ϕ* is:

(5) $$\frac{d\phi }{dt}=\left(s+r\phi +k\phi^{2}\right)\left(1-\phi \right)-u\phi$$

**Figure 2:**
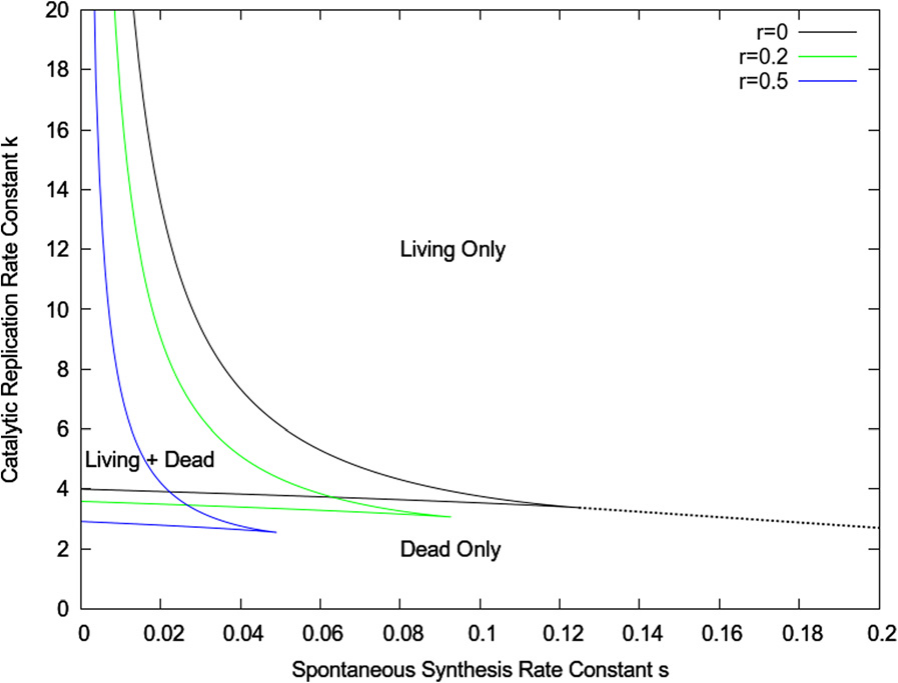
**Phase diagram for the simple replicator model (Eqn. 5) as function of catalytic feedback efficiency *k* and spontaneous synthesis rate constant *s* with *u*= 1.** Boundaries are shown for three different values of *r*.

In many models of population dynamics in evolution and ecology, the linear growth term, *rϕ*, is natural, and the spontaneous term, *s*, and nonlinear term, *kϕ*^*2*^ are not considered. However, when considering the origin of life, the spontaneous term is essential for generation of the initial replicators, and the nonlinear term is essential in order to give the possibility of two stable states. The linear term is less important; therefore we will first consider the case where *r* = 0. The stationary states are the roots of this cubic equation. If *s* is small and *k* is fairly large, there are two stable states, *ϕ←*_*1*_ and *ϕ*_*2*_, separated by an unstable state *ϕ*_*3*_. The lower stable state is a dead state, controlled by the balance between spontaneous generation and death: *ϕ∼s*/*u*. The upper stable state is a living state controlled by the catalytic term *k*. If *k* is large, the concentration will approach the carrying capacity, *ϕ*_*2*_*∼*1. It is easy to obtain phase diagram for existence of dead and living states as in Figure 2. This has the same regions as Figure 1. If the linear growth term *r,* is non-zero, the positions of the phase boundaries move, but shape of the diagram is qualitatively unchanged (also shown in Figure 2). We therefore argue that this kind of phase diagram is a generic feature of replicator models for the origin of life, and that Equation (5) is the simplest possible dynamical equation that has this phase diagram.

### The two’s company model

The main aim of this paper is to consider the influence of spatial concentration fluctuations and limited diffusion rate on the stochastic transition that leads to life. Therefore, we will now define a spatial lattice model, which we call the Two’s Company Model, that is a spatial version of Eqn. 5, and reduces to Eqn. 5 when the system is well mixed.

**Figure 3:**
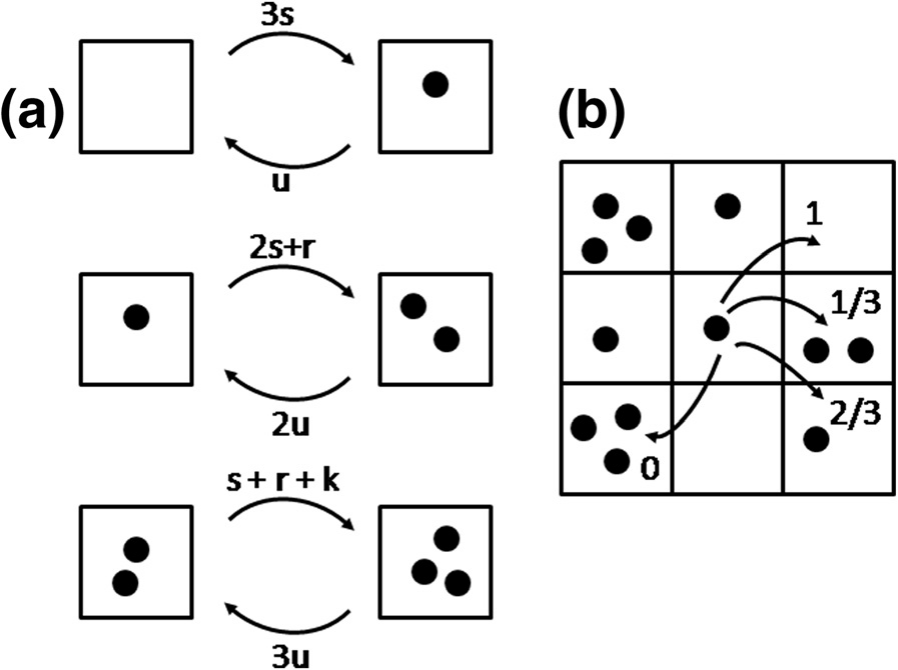
**(a) Transition rates in the Two’s Company model due to birth and death events on a single site.** (**b**) Each molecule attempts to hop to a neighbouring site at rate *h* and is successful with a probability equal to the fraction of vacancies on the neighbour.

We consider a 2D square lattice of *L×L* sites. The number of molecules on any one site may be *n* = 0, 1, 2 or 3 only. The carrying capacity of the whole lattice is therefore 3*L*^*2*^. If there are *N* molecules on the lattice, the mean concentration relative to the carrying capacity is *ϕ = N/*(3*L*^*2*^). There are 3-*n* vacancies on a site with *n* molecules. Vacancies are treated as resources, and the rates of spontaneous, linear, and replicative growth are all proportional to the number of vacancies. The rate of adding a molecule by the spontaneous reaction is defined as *s* times the number of resources, *i.e. s*(3-*n*). The rate of adding a molecule by the linear growth process is defined as *r*/2 times the number of molecules times the number of resources, *i.e. rn*(3 − *n*)/2. This is equal to *r* when *n* = 1 or 2, and zero otherwise. The rate of catalytic replication is defined as *k/2* times the number of ways of picking a replicator-template pair times the number of resources, *i.e. n*(*n* − 1)(3 − *n*)*k*/2. This is *k* when *n* = 2 and zero otherwise. The replication process in the model can only occur when *n* = 2. “Two’s company, three’s a crowd”; hence the name of the model. Combining the three birth processes, we obtain the total birth rate of molecules on a site with *n* molecules, as summarized in Figure 3. The death rate of a molecule on a site with *n* molecules is *un* (i.e. *u* per molecule).

Hopping of molecules between sites is implemented using either local or a global hopping rules in the following way. Each molecule attempts to hop at rate *h*. A destination site is chosen for the molecule. In the case of local hopping rules, the destination is chosen at random from the 8 neighbouring sites of the original site. In the case of global hopping rules, the destination site is chosen at random from all the other sites on the lattice. The molecule hops successfully to the destination site if it finds a vacancy there, *i.e.* the hop is successful with probability 1-*n*/3 (as shown in Figure 3). If the hop is unsuccessful, the molecule remains on its original site. Further details of the stochastic dynamics used to implement the model are given in the Methods section.

The local hopping rules simulate local molecular diffusion, which is an important part of this model. The global hopping rules are intended as a comparison that helps to illustrate the importance of local hopping. The introduction of local hopping rules means that correlations exist between the numbers of molecules on neighbouring sites. However, if *h* is sufficiently large, the system reaches a well-mixed limit where molecules are randomly positioned over the whole lattice and there is no remaining correlation between sites. In the case of global hopping, there is no correlation between sites, even when *h* is small. Below, we will discuss the mean field approximation, which is exact for the global hopping dynamics but it is only an approximation for the model with local hopping dynamics. In the Methods section, we show that when *h* is very large, both global and local hopping models reach the same well-mixed limit, which corresponds to the generic replicator model in Equation 5.

Simulations are initiated in the dead state and followed until a transition to the living state occurs. The concentration in the dead state should be close to the stable solution *ϕ*_*1*_ of Equation 5. In order to initiate the stochastic simulation in the dead state, each lattice site is seeded with molecular numbers sampled from a binomial distribution with average concentration *ϕ*_*1*_. In a typical simulation, the system remains in the dead state for a long time until a localized patch of high concentration arises that is sufficiently large to be stable in the living state. The origin of this living patch is a rare stochastic event. However, once it is formed, the living patch then spreads deterministically across the whole lattice.

**Figure 4:**
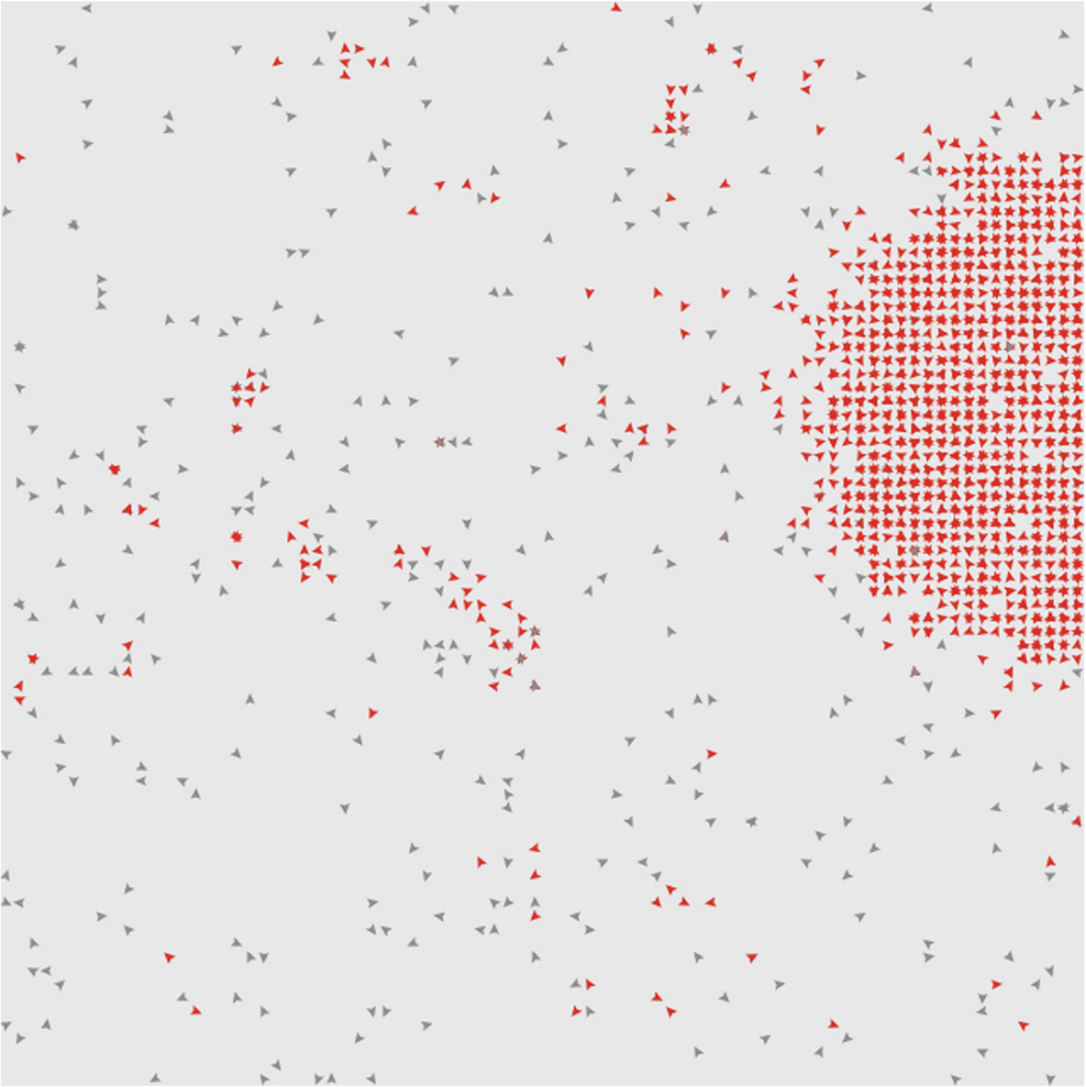
**Snapshot of a simulation of the Two’s Company model shortly after the transition to life.** The living state is a dense patch of replicators that have been synthesized catalytically (red). The non-living state is characterized by a low density of replicators created by spontaneous synthesis (coloured grey) with small isolated denser patches. Once the living patch is big enough to be stable, it spreads deterministically across the lattice.

A snapshot of a simulation shortly after the transition to life is shown in Figure 4. Molecules that were synthesized by the spontaneous process are coloured grey, and molecules that were synthesized by the catalytic process are coloured red. These colours are used for illustration, but all the molecules have identical properties. In Figure 4, a living patch has arisen in one region. Within this region, there is a high concentration (most sites have *n =* 2 or 3), and most molecules are red, indicating that this patch is sustained by catalytic replication. The rest of the lattice is still in the dead state, where there is a low concentration (most sites have *n =* 0 or 1), and most molecules are grey. Small clusters of red molecules are nevertheless visible within the dead region. These clusters appear and disappear rapidly. The origin of life occurs when a cluster of this kind becomes large enough to be stable and to continue to grow. If the spontaneous rate *s* is suddenly switched to zero in the configuration shown in Figure 4, then the isolated molecules in the dead state rapidly disappear, but the living patch remains virtually unaffected and continues to spread. The spontaneous process is not necessary to sustain the living state because the living state is autocatalytic. Of course, the spontaneous rate must be non-zero in order to allow the transition to the living state in the first place.

We performed many simulations of this model in order to measure the way the time taken for the transition depends on the parameters. We define *T*_*sys*_ = *T*_*reg*_ + *T*_*spread*_, where *T*_*reg*_ is the time until a local transition occurs in any one region of the lattice, and *T*_*spread*_ is the time for the living state to spread from one region across the rest of the lattice, and *T*_*sys*_ is the time required for the full lattice to reach the living state. These times were measured as described in the Methods section.

**Figure 5:**
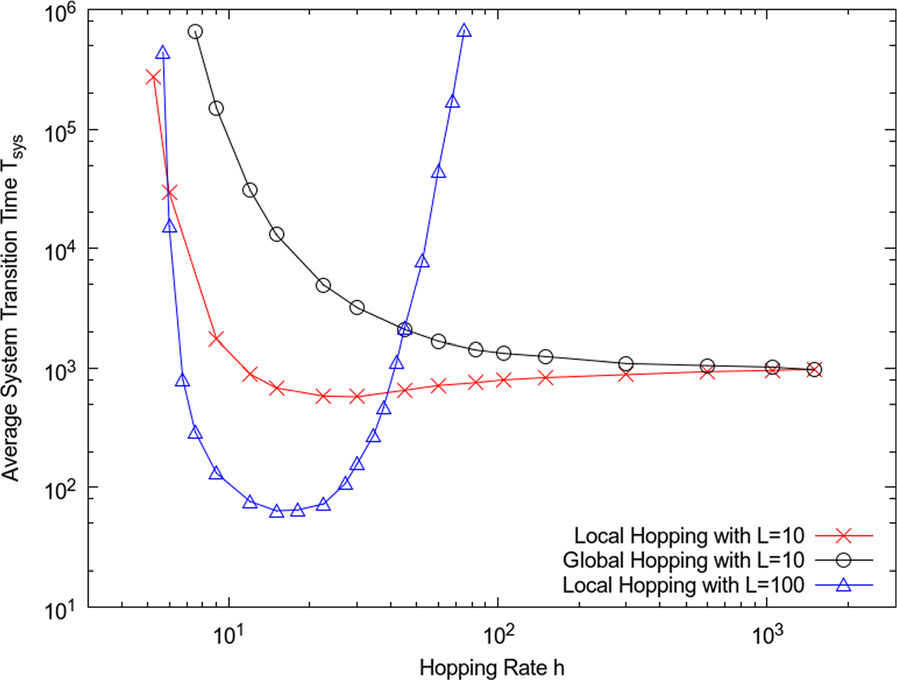
Effect of hopping rate on transition time for fixed system size with other parameters set as: *s*=0.02, *r*=0, *k*=9.

Figure 5 shows the average *T*_*sys*_ as *h* is varied with a fixed system size. A comparison of the transition times with local and global hopping rules is shown for a small lattice with *L* = 10. For large *h* the measured times for local and global hopping converge, showing that the system is well mixed. For smaller *h*, the transition time with local hopping is always shorter than that with global hopping. This shows that the local concentration fluctuations that arise in the local hopping model make the transition much easier. It can be seen that if *h* is very small, the transition time becomes very long, both for local and global hopping. It should be remembered that the spontaneous generation rate *s* is very small, so the concentration of molecules in the dead state is very small. For the catalytic process to occur, it requires two molecules on the same site. If *h* is too small, molecules do not encounter one another frequently. Furthermore, if molecules do encounter one another and a replication occurs, there will now be three molecules on one site, which blocks further replication. It is necessary for one of these molecules to hop away before a second replication can occur; hence, *h* should not be too small. It can be seen that, for the local hopping rules, there is an optimum diffusion rate at which the transition is fastest. This effect is not very strong in the small lattice (*L* = 10), but is very pronounced in the larger lattice (*L =* 100) also shown in Figure 5. The transition time decreases by several orders of magnitude in the middle of the range of *h*. It is not possible to show the transition time for the global hopping case for the larger lattice size because it is too long to measure accurately in the simulation with these parameters. In summary, Figure 5 shows that local diffusion is essential in order to allow the transition to life to occur at a reasonable rate in large systems, and when diffusion is local, there is an optimal intermediate rate of diffusion.

**Figure 6:**
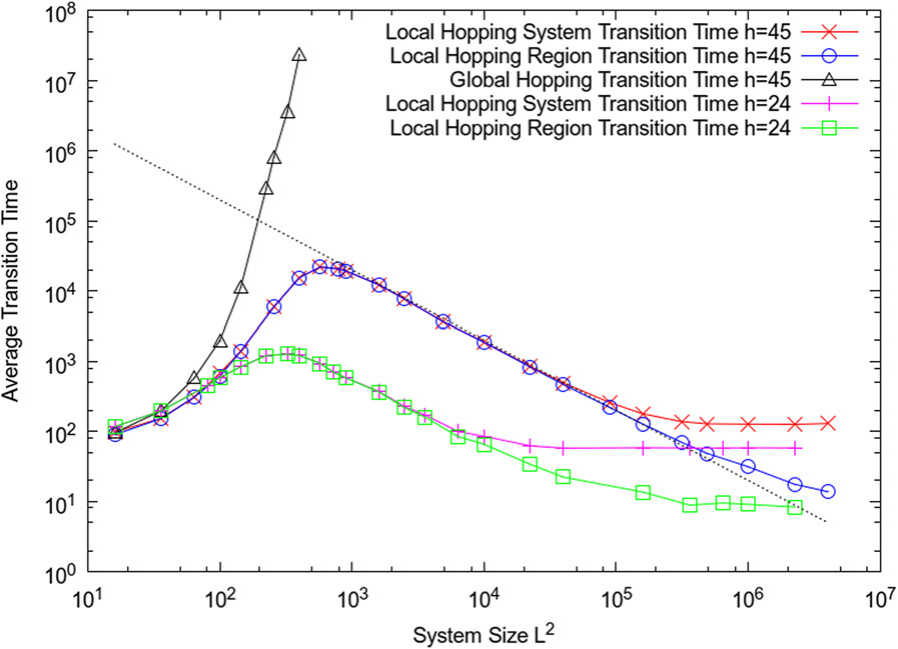
**Average transition time from dead state to living state as function of system size with parameters set as: *s*=0.02, *r*=0, *k*=9, *u*=1.** The straight line illustrates a scaling of 1/*L*^*2*^.

Figure 6 shows the variation of transition time with system size when the hopping rate is kept fixed. For the global hopping case, it is only possible to do these simulations for fairly small lattice sizes, because the transition time increases very rapidly with *L*. For the local hopping case, it is possible to carry out simulations over a much wider range of *L*, and it can be seen that the transition time actually decreases with *L*, if *L* is sufficiently large.

As shown in left part of Figure 6, when the system is small, it is more or less well mixed. Hence the transition will happen globally across the whole system at the same time. When the system is larger, it is no longer homogeneous. The transition will happen at a local region first, then it will spread out to the whole system. The number of independent local regions should be proportional to *L*^*2*^. The average time taken for the transition to occur in any one of these regions should vary inversely with the number of regions, *i.e. T*_*reg*_*~ 1/L*^*2*^*.* If the spread of the living state is rapid, the spreading time, *T*_*spread*_ is small compared with the time taken for the transition to occur, *T*_*reg*_. In this case the time taken for the whole system to go through the transition is close to *T*_*reg*_. It can be seen that there is an intermediate range of *L* in Figure 6, where *T*_*reg*_ and *T*_*sys*_ are very close to one another and where both scale as 1/*L*^*2*^, as expected from this argument. Comparison of the results with *h* = 24 and *h =* 45 shows that when diffusion is slower, the transition from homogeneous system to heterogeneous system happens for a smaller system size.

If the system size is extremely large, there comes a point where *T*_*spread*_ is comparable to *T*_*reg*_, and the curves for *T*_*sys*_ and *T*_*reg*_ separate in Figure 6. At this point it is possible for the transition to life to occur in a second region independently before the living state from the first transition has spread across the lattice. Beyond this point, *T*_*reg*_ continues to decrease, but *T*_*sys*_ reaches a constant value, which is what we expect if there are multiple origins happening in different places.

A useful way to understand the effects of the limited diffusion rate in this model is to calculate the mean field solution. In the Methods section, we calculate the probability *P*_*n*_ that there are *n* molecules on a site, using a mean field approximation that ignores correlations between neighbouring sites. The mean field solution depends on *h* and it is not equivalent to the well-mixed case, except for the limit of large *h*, where both the lattice model and the mean field approximation converge to the well-mixed case. We expect that the mean field approximation will be fairly good for the local hopping model if *h* is large, because correlations between neighbouring sites should be small in this limit. For the global hopping model, the mean field solution is exact, even for small *h*.

**Figure 7:**
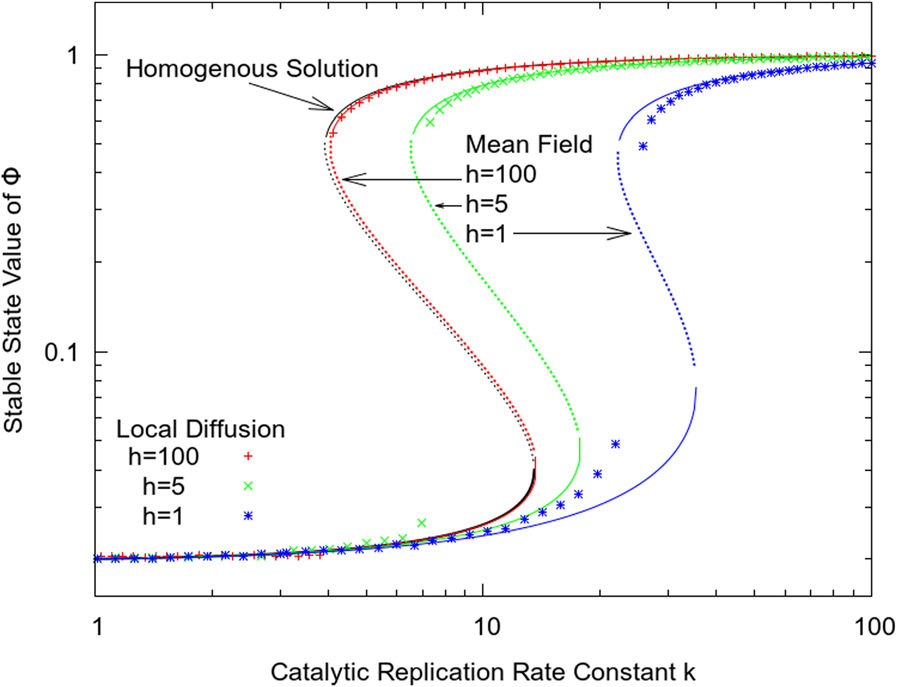
**Comparison of stable state solution between local diffusion model, mean field theory and homogeneous model as function of replication rate *k*.** Other parameters are set as: *s*=0.02, *r*=0, *u*=1, *L*=100.

In Figure 7, we compare the solutions of the mean field equations with dynamical simulations. The black curve is the homogeneous solution from the roots of the cubic Equation 5. This is the well mixed limit of the spatial model. The solid black lines illustrate the stable living and dead states and the dashed line is the unstable intermediate state. The red, green and blue lines show the stable and unstable solutions of the mean field equations with three values of *h*. For large *h*, the mean field solution is close to the well-mixed limit, as expected. The symbols show average concentrations of molecules obtained from simulations of the lattice model with local hopping. For the living state, the mean field theory gives a fairly good approximation to the result with local hopping, even for the smaller values of *h*. This is because the density is quite high across the whole lattice in the living state and correlations between neighbouring sites are quite weak. On the other hand, there are large deviations between the mean field theory and the results of the local hopping model in the dead state when it is close to the bistable region, which shows that there are important local spatial correlations in this case.

Figure 7 illustrates that reduction in the local diffusion rate has both positive and negative effects on the replication process. As *h* is reduced, the concentration of the living state is reduced because there is more local interference between molecules on fully occupied sites. Also the minimum value of *k* required for the living state to be stable increases as *h* decreases. Thus, rapid diffusion is favourable for the living state, if the living state is already established. However, we have already seen in Figure 5 that high diffusion rates cause the system to be well mixed, in which case the transition time is extremely long. It is therefore important to have a diffusion rate that is not too large in order for the transition to the living state to occur at an appreciable rate in the first place.

### A spatial model for RNA polymerization

The Two’s Company model is the simplest model that can be used to study the stochastic transition to life in a spatially distributed system. However, we wish to show that the same behaviour occurs in other models. We will return to the model of RNA polymerization described in Equations 1–4 in order to study the effects of diffusion and spatial concentration fluctuations in this case.

The simulation is carried out on 2D square lattice composed of *L* × *L* sites, with each site defined to have a size *l* = 1. We keep track of how many molecules of each kind there are on each site. The reactions of monomer synthesis, activation, and polymerization all occur locally on individual lattice sites. The implementation of stochastic dynamics for this model using the Gillespie algorithm is described in our previous paper [26] for a non-spatial model. In the spatial case, the same method is used to define reaction rates on each site as a function of the numbers of molecules of each type on each site. For simplicity, molecules of all kinds are assumed to hop with the same rate *h*. Hopping may either be local or global, as with the Two’s Company model. However, there is no need to impose a maximum number of molecules per site, because concentration is limited by food input rather than by a carrying capacity. Therefore every attempted hop is successful, and hops are not blocked by the molecules on the destination site, as they are for the Two’s Company model.

**Figure 8:**
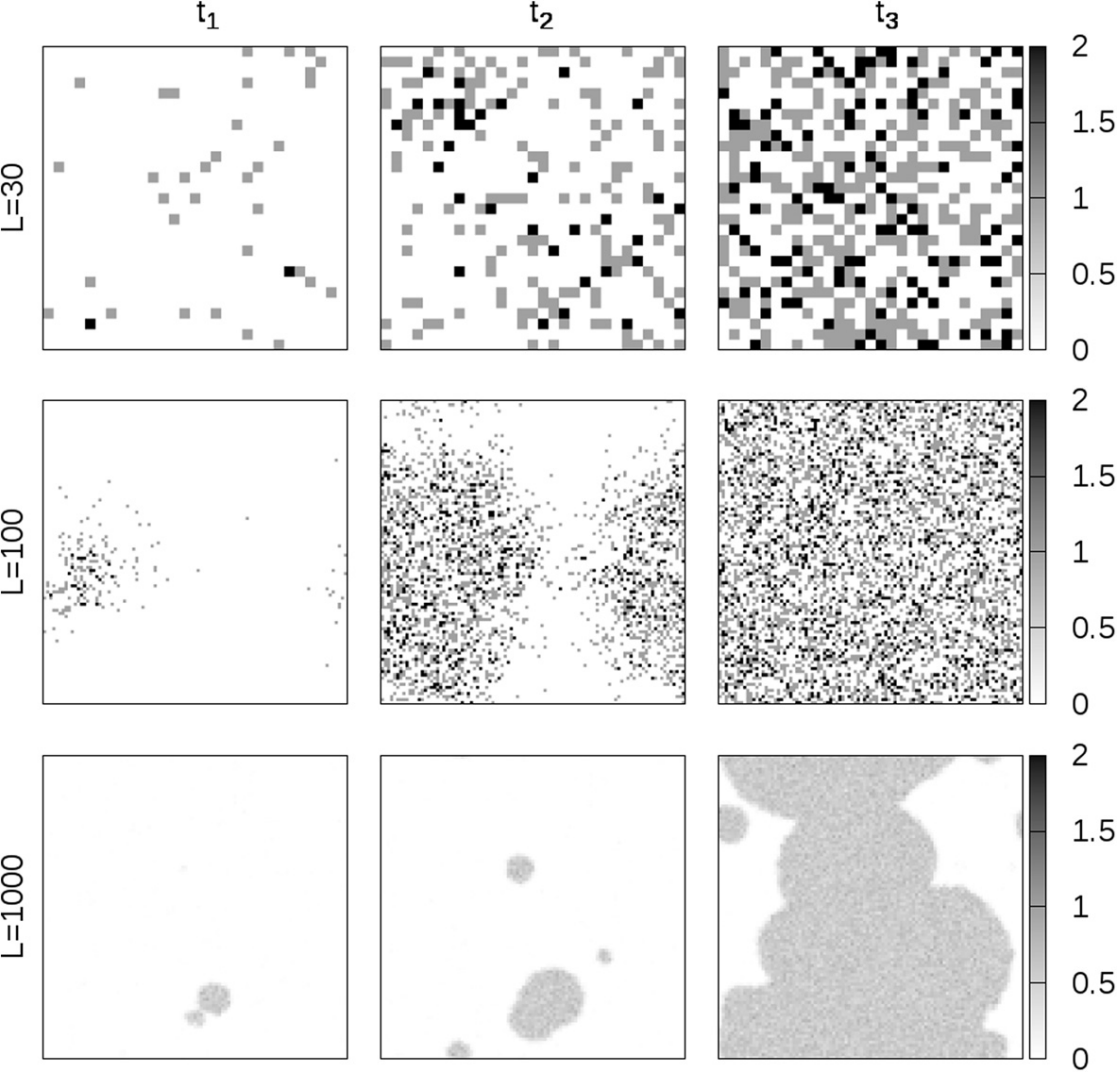
**Snapshots of the catalytic polymer concentration in the RNA polymerization model at the beginning(*t*_1_), during(*t*_2_), and after(*t*_3_) the transition from the dead state to the living state for different system sizes (L=30, 100, 1000) and with local hopping rules.** Parameters are set as: *m*=5, *s*=10, *a*=10, *r*_0_=0.02, *k*=2, *u*=1, *h*=50, *f*=1.

Figure 8 shows snapshots of the distribution of catalytic polymers across the lattice. Three time points are chosen in order to illustrate the way the catalyst concentration increases over time. As shown in first row of Figure 8, when system is small (*L*=30), the system is fairly well mixed. Hence the transition happens across the whole system at the same time. When system is larger (*L*=100), the system is no longer homogeneous. The transition happens at a local region first, then it spreads out to the whole system as shown in second row of Figure 8. When the system is very large (*L*=1000), the time it takes to spread across the whole system is longer than the regional transition time. Hence, multiple origins occur, as in third row of Figure 8.

When the transition occurs, the increase in the catalyst concentration is accompanied by a sharp decrease in the activated monomer concentration. As there are many more activated monomers than catalysts, the activated monomer concentration varies more smoothly than the catalyst concentration. Therefore, we used the decrease in the activated monomer concentration as a marker for the transition time. This concentration was averaged over a sliding time interval, and this was used to define regional and system transition times, in the same way as for the Two’s Company model (see Methods section).

**Figure 9:**
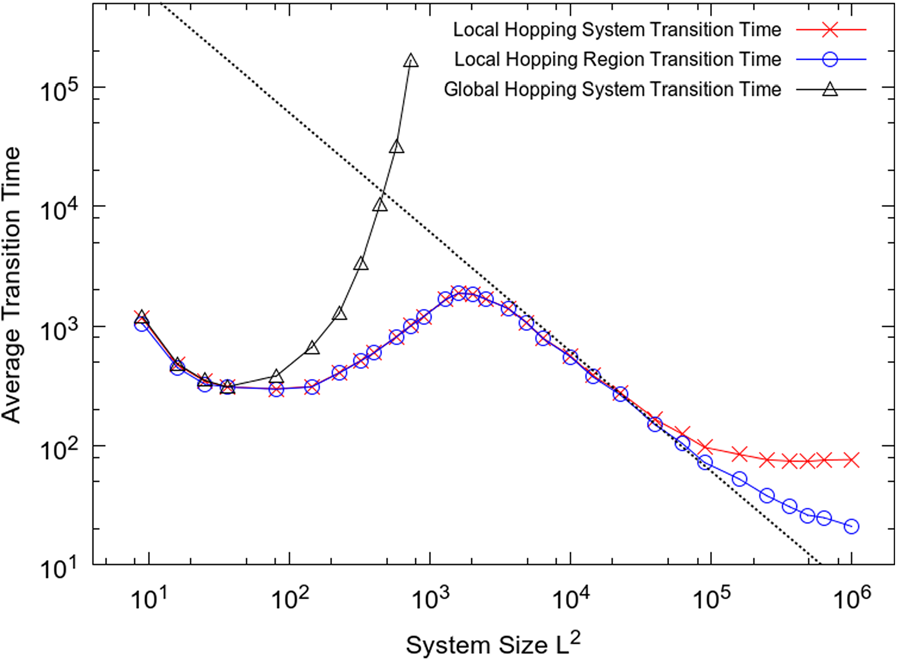
**Average transition time from dead state to living state as function of system size with parameters set as: *m*=5, *s*=10, *a*=10, *r*_0_=0.02, *k*=2, *u*=1, *h*=50, *f*=1.** The straight line illustrates a scaling of 1/*L*^*2*^.

The transition time is shown as function of system size in Figure 9. For global hopping, the transition time follows a U-shaped curve, for the same reasons as for the non-spatial model of RNA polymerization (Figure seven of [26]). If the system is too small, polymers long enough to be catalysts do not easily arise, and if the system is too big, the concentration fluctuations are too small in the global case to allow the stochastic transition to occur. In contrast, when hopping is local, the transition occurs easily for large system sizes. When the lattice is very small, the time for the transition with local hopping is close to that with global hopping, but when the lattice size is larger, the transition is much faster with local hopping. The transition time follows the same shape curve for this model as for the Two’s Company model in Figure 6. There is an intermediate range where the transition time scales as 1/*L*^*2*^ and where *T*_*sys*_*= T*_*reg*_, and there is a range for very large lattice size where multiple origins occur and where *T*_*sys*_*> T*_*reg*_.

## Discussion and conclusions

The models we have discussed here have features in common with various theoretical models discussed by other authors. There has been a lot of work on maintenance of replicators in the face of accumulation of mutations. The standard error threshold problem [33] deals with linear replicators in a non-spatial model. A spatial lattice version of this problem has been studied [33] and this has been compared to the case of catalytic replicators in a spatial system [34] using a model similar to the Two’s Company model. The fundamental difference between linear and catalytic replicators is that when a replicator acts on another sequence as template it is being altruistic. Evolution of catalytic replicators is therefore related to the evolution of cooperation. A large number of studies have shown that cooperation can evolve in spatial models when the equivalent non-spatial models would be invaded by parasites [35-43]. These models make it clear that formation of clusters of cooperating molecules and formation of spatial patterns is generally very important for evolution and survival of catalytic replicators. However, none of these papers dealt with the origin of the replicating system. Our work also shows that incorporation of local diffusion and spatial concentration fluctuations is essential to enable the origin of life to occur with an appreciable probability. Other models [44-46] that deal with prebiotic replicators and consider the transition to a replicating state are closer to our work. Spatial effects are also apparent in these models. Another approach to the origin of the RNA world [47] uses very complex sets of reaction equations for the emergence of RNA replication and also makes a link with nucleation phenomena. Models for the origin of replication [29-31] have been studied previously that are very similar to ours, however the stochastic dynamics and spatial effects were not looked at by these authors.

Despite important differences in their details, the Two’s Company model and the RNA polymerization models exhibit surprisingly similar behaviour, both for the phase diagram (Figures 1 and 2) and the dynamics of the transition to life (Figures 6 and 9). The similarity arises because the models share the features of nonlinear feedback and limited resources. For the Two’s Company model, the replication rate of the molecule depends nonlinearly on the concentration of the molecule because of the *kϕ*^*2*^ term, and limited resources are modelled by the rule that there can be no more than 3 molecules on the same site. For the RNA polymerization model, the nonlinearity arises because of the feedback in polymerization (Eqn. 4) because of the way the ribozyme concentration depends on the polymerization rate (see [26]). The limited supply of food molecules from the environment is included in the model, and this prevents the ribozyme concentration increasing indefinitely in the living state. Given these two features, both models have two stable states and a bistable region in the phase diagram.

The two models exhibit essentially the same stochastic transition arising as a result of concentration fluctuations. When the system is well mixed, fluctuation will decrease with increasing system size due to increasing number of molecules in the system. Hence the transition becomes increasingly difficult for larger system sizes. With limited diffusion, the system is no longer well mixed and breaks down into many weakly interacting regions. The larger the system, the more such independent regions there are in which the transition can occur. Therefore the transition becomes faster for larger systems in a 2D model with limited diffusion.

Both models illustrate that multiple origins of life could occur if the system is large enough. As far as we know, however, all current life on Earth is descended from a single origin. The size of the Earth is very much larger than the distance over which a molecule could easily diffuse. However, the window of time during which life arose is narrowed to a few hundred million years by geological evidence [48]. If the origin of life is a rare event taking hundreds of millions of years, it is still perfectly possible that it occurs only once (or not at all) on a large planet. Once the living state has arisen, the time for multiplication and spread across the planet is determined by molecular time scales (replication and diffusion) which are likely to be very much faster than the waiting time for the rare event that creates life in the first place. In other words, it is likely that the real world falls in the intermediate part of Figure 9, where the transition time varies like 1/*L*^*2*^ and there is a single origin. However, the scenario involving multiple origins could still be compatible with the view of single tree of life if there were competition between different forms of life and only one remained, or if there were symbiosis and merging of features that derived from different origins.

Two parameters that are important in the RNA polymerization model are *m*, the minimum sequence length required to be a catalyst, and *f*, the probability that a sequence of length *n ≥ m* is a catalyst. In this paper, we ran many simulations for each set of parameters in order to study the way the mean transition time depended on *h* and *L*. For this reason, it was necessary to choose parameters for which the transition occurred fairly easily. Therefore, we made the length short (*m = 5*) and chose the easiest case where *f =* 1. In our first paper, we looked at much longer ribozymes (*m =* 50) with *f* much less than 1, which seems more realistic if we are looking for ribozymes that would have specific sequences and structures, and the essential behaviour is very similar. In reality, we do not know how long the first catalytic sequences might have been. We are used to thinking of enzymes hundreds of amino acids long in current organisms, and currently known polymerase ribozymes are all well over 100 nucleotides long [7,9,14]. Hence, we tend to think of biopolymers as rather long and very specific in their sequence. It seems reasonable to suppose that the earliest catalysts must have been much shorter and less sequence specific if they were to have had any chance of arising in prebiotic conditions. Short sequences would be relative common, and relatively easy to replicate even if the fidelity was quite low. These are important advantages of short sequences, even if they have only very limited catalytic ability compared to much longer, well-adapted ribozymes.

In all our models of RNA polymerization, we have made the simplification of ignoring nucleotide sequences and keeping track only of polymer length. It is supposed that polymerase ribozymes catalyze all polymerization reactions equally. Hence, they will catalyze the formation of random non-functional sequences in addition to increasing their own formation rate. This would be extremely inefficient, and it seems likely that real ribozymes could have done better than this by being more specific. Nevertheless, an autocatalytic living state arises even in this simple non-specific case, and there is a clear difference between the living and the dead state, which is the aspect of the model we wish to emphasize. The case of non-specific polymerases has sometimes been called “pre-life” [29-31], and the term “life” has been reserved for a specific polymerase that catalyzes only the steps that lead to formation of its own sequence. Such a specific polymerase would have been much more efficient, as it would not waste its time making non-functional polymers. However, it is difficult to see how a specific polymerase would distinguish between the polymerization steps that contribute to its own formation and those that do not. In fact, polymerases in modern organisms are general, not specific. They can synthesize any general sequence, but they make specific sequences because they make use of templates, not because they distinguish between the reaction steps that link different kinds of monomers.

Of course, not all sequences are equivalent in the real world, and whether a molecule can function as a catalyst depends on its sequence and structure. It would be possible to consider a model for polymerization of four nucleotides in which the base sequence of each polymer in the mixture is stored in memory. One could then determine whether a sequence was a catalyst using criteria based on secondary structure or whether it contained a specific sequence motif within it. This would be more realistic than simply giving each sequence a probability *f* of being a catalyst, as in our current model, but the essential point that the formation of catalysts causes a feedback in the polymerization rate would remain unchanged in a more complex model that considered RNA sequence and structure in more detail. However complex and realistic we try to make a model, there will always be factors that must be left out. In some cases, simplicity is a virtue in computational models if this helps to show the essential points clearly. We believe that the simple models studied here illustrate several essential features of the origin of life problem as it occurred in the real world, *i.e.* the origin of life is a transition between two alternative steady states of a chemical reaction system that is driven by stochastic concentration fluctuations involving relatively small numbers of molecules in a localized region of space.

## Methods

We simulated the stochastic dynamics of the Two’s Company Model using the Gillespie algorithm. At each time step, one elementary reaction was chosen at random with a probability proportional to its rate. The duration of the time step was a random time *τ*, chosen from an exponential distribution *P*(*τ*) = Re^− *Rτ*^, where *R* is the sum of the rates of all the elementary reactions that could occur at that point in time.

Simulations were performed to measure the mean time until the transition to the living state occurred. The simulations were stopped when the global concentration became close to *ϕ*_*2*_, the solution of Equation 5 corresponding to the living state. In order to insure that the system was stable in the living state, *ϕ* was averaged over a sliding time interval of width 20, which is 20 times the average life time of a molecule. The simulation was stopped when the sliding average density reached 90% of *ϕ*_*2*_. This time was then recorded as the system transition time of one trial. The average system transition time, *T*_*sys*,_ was obtained from 1000 trials for each set of parameters. When the local hopping rule is used, the transition happens at one place then spreads across the whole system. We may write *T*_*sys*_*= T*_*reg*_ + *T*_*spread*_ , where *T*_*reg*_ is the time until a local transition occurs in any one region, and *T*_*spread*_ is the time for the living state to spread from one region across the rest of the lattice. The system was divided into multiple regions of size 10×10 lattice sites. The concentration was calculated within each region and averaged over a time window in the same way as the global concentration. *T*_*reg*_ was defined as the point at which the sliding window concentration of any one region reached 90% of *ϕ*_*2*_*.*

We will now demonstrate that the well mixed limit of the lattice model corresponds to the generic replicator model in Equation 5. The total birth rate of molecules, *a*_*n*_, on a site with *n* molecules is

(6) $$\begin{array}{l} a_{0}=3s\\ a_{1}=2s+r\\ a_{2}=s+r+k \end{array}$$

The death rate of a molecule on a site with *n* molecules is *nu*. Let *P*_*n*_ be the fraction of sites that have *n* molecules. The rate of change of *N* due to birth and death processes is

(7) $$\frac{dN}{dt}=L^{2}\left(a_{0}P_{0}+\left(a_{1}-u\right)P_{1}+\left(a_{2}-2u\right)P_{2}-3uP_{3}\right)$$

Hence

(8) $$\frac{d\phi }{dt}=\frac{1}{3}\left(a_{0}P_{0}+\left(a_{1}-u\right)P_{1}+\left(a_{2}-2u\right)P_{2}-3uP_{3}\right)\text{.}$$

Note that these equations do not depend on the hopping rate because the hopping process does not change the total number of molecules. However, the *P*_*n*_ probabilities do depend on *h*. If both *N* and *L* are large and *h* is large enough to be in the well-mixed limit, where molecules are randomly distributed between lattice sites, *P*_*n*_ has a binomial distribution:

(9) $$P_{n}=\phi^{n}{\left(1-\phi \right)}^{3-n}\frac{3!}{n!\left(3-n\right)!}$$

Substitution of these probabilities into Equation 8 gives Equation 5. Thus, the lattice model reduces to the desired continuum model if there is rapid mixing of molecules.

We now consider the rates of gain and loss of molecules due to molecules hopping to and from a site with *n* molecules. Let *q*_*nm*_ be the conditional probability that there are *m* particles on the neighbouring site, given that there are *n* on the first site. The rate that *n* increases by 1 due to a molecule arriving from a neighbouring site is

(10) $$p_{n}^{+}=h\left(q_{n1}+2q_{n2}+3q_{n3}\right)\left(1-\frac{n}{3}\right)=3\left(1-\frac{n}{3}\right)h\phi_{n}\text{,}$$

where *ϕ*_*n*_ = (*q*_*n*1_ + 2*q*_*n*2_ + 3*q*_*n*3_)/3 is the mean density on sites that are neighbours of sites with *n* particles. Note that there are 8 neighbours from which a molecule could come, but only 1/8 of the hops from any one neighbour move to the site in question. Therefore these two factors of 8 cancel out.

The rate at which *n* decreases by 1 due to a molecule leaving the site is

(11) $$\begin{array}{l} p_{n}^{-}=hn\left(\left(1-\frac{1}{3}\right)q_{n1}+\left(1-\frac{2}{3}\right)q_{n2}+\left(1-1\right)q_{n3}\right)\\ \;=hn\left(1-\phi_{n}\right)\text{,} \end{array}$$

We may now write a set of equations for the rates of change of *P*_*n*_ that include birth, death and hopping.

(12) $$\begin{array}{l} \frac{dP_{0}}{dt}=-a_{0}P_{0}+uP_{1}+p_{1}^{-}P_{1}-p_{0}^{+}P_{0}\\ \frac{dP_{1}}{dt}=a_{0}P_{0}-a_{1}P_{1}+u(2P_{2}-P_{1})+p_{2}^{-}P_{2}-p_{1}^{+}P_{1}\\ \;-p_{1}^{-}P_{1}+p_{0}^{+}P_{0}\\ \frac{dP_{2}}{dt}=a_{1}P_{1}-a_{2}P_{2}+u(3P_{3}-2P_{2})+p_{3}^{-}P_{3}\\ \;-p_{2}^{+}P_{2}-p_{2}^{-}P_{2}+p_{1}^{+}P_{1}\\ \frac{dP_{3}}{dt}=a_{2}P_{2}-3uP_{3}-p_{3}^{-}P_{3}+p_{2}^{+}P_{2} \end{array}$$

This set of equations is not closed, because the hopping rates depend on the conditional probabilities *q*_*nm*_. A standard way of approximating the solution to lattice models is to make a mean field approximation that ignores correlations between neighbours. We assume that *q*_*nm*_*= P*_*m*_, in which case:

(13) $$\phi_{n}=\phi =\frac{1}{3}P_{1}+\frac{2}{3}P_{2}+P_{3}$$

With this approximation, we get the following equations, which are a closed mean field approximation to the dynamics.

(14) $$\begin{array}{l} \frac{dP_{0}}{dt}=-3sP_{0}+uP_{1}+h((1-\phi )P_{1}-3\phi P_{0})\\ \frac{dP_{1}}{dt}=3sP_{0}-(2s+r)P_{1}+u(2P_{2}-P_{1})\\ \;+h(2(1-\phi )P_{2}-2\phi P_{1}-(1-\phi )P_{1}+3\phi P_{0})\\ \frac{dP_{2}}{dt}=(2s+r)P_{1}-(s+r+k)P_{2}+u(3P_{3}-2P_{2})\\ \;+h(3(1-\phi )P_{3}-\phi P_{2}-2(1-\phi )P_{2}+2\phi P_{1})\\ \frac{dP_{3}}{dt}=(s+r+k)P_{2}-3uP_{3}\\ \;+h(-3(1-\phi )P_{3}+\phi P_{2}) \end{array}$$

The mean field solutions shown in Figure 7 are the stationary states of these equations. The simulation with global hopping rules follows the mean field solution exactly because there are no correlations between sites when hopping is random. The mean field solution is an approximate solution for the case with local hopping rules.

## Competing interests

The authors declare that they have no competing interests.

## Authors’ contributions

MW designed the models, carried out numerical analysis and simulations of the RNA polymerization models and wrote the paper. PH designed the models, carried out simulations of the Two’s Company model and wrote the paper. Both authors read and approved the final manuscript.

## Reviewers’ reports

Reviewer 1: Omer Markovitch, Weizmann Institute of Science (nominated by Doron Lancet).

The authors argue that a phase diagram, exhibiting living and non-living states (when the templating rate is respectively bigger or equal to the spontaneous polymerization rate), is a generic feature of replicator-first origin of life models. The authors then develop a simple lattice model in which a site can contain up to 3 molecules, templating can occur when exactly 2 monomers exist in a site and diffusion between adjacent sites is allowed. Their spatial model exhibits patches of replicating molecules, some of which can eventually grow to dominate the system akin to a percolation transition, in analogy to a bistable diagram. They find that rapid diffusion in a large lattice inhibits the non-life to life transition because fluctuations are smaller. The manuscript deals with spatial effects in replicating polymers, which did not receive full attention before, and presents the results in a clear manner, thus deserves publication.

The discussion of 3-molecules-limit vs. no-limit is suggested to be extended. The authors should consider performing an intermediate test in order to verify that carrying capacity is qualitatively similar to concentration limit.

Response: *We would not call this a percolation transition. The analogy we are making is with a nucleation phenomenon, such as nucleation of solid crystals in a liquid followed by subsequent rapid growth of the crystals.*

In the RNA polymerization model, resources are explicitly modelled, and therefore no carrying capacity is necessary. In the Two’s Company it is necessary to impose a carrying capacity in order to avoid unbounded growth. It would also be possible to add a resource molecule explicitly to the Two’s Company model, but this would go against our intention of making this model as simple as possible.

1) The abstract will benefit from being toned down, example given from “The key step is…” to “A key step is…”.

Response: *We changed this to A in the abstract.*

2) Providing information on the existence of alternative views to the RNA world is suggested.

Response: *We added citations to Shapiro [*[23]*] and Segre and Lancet [*[24]*] in the introduction.*

3) Section 2 (A Generic Phase Diagram for Replicating Molecules) is too long. It is suggested to provide fewer details of a previously published work and stress the stress the conclusion right at the beginning.

Response: *We have made only very slight reductions in this section. All the equations are necessary for what follows in this paper, and the textual discussion is important. The first two sentences already state the purpose and conclusion of this section.*

4) The reader could benefit from an illustration / diagram of the “Two is a company” model.

Response: *A new Figure*3*has been added showing the rules for this model.*

Reviewer 2: Claus Wilke, University of Texas, Austin.

Comment: This article uses mathematical modeling and computer simulations to study a simple origin-of-life model. The two main contributions this article makes are (i) a simplified model of the origin of life that maps in a straightforward fashion to a more complex model and (ii) a detailed analysis of the origin-of-life model in a spatial setting. The authors address the conditions under which a living state can emerge from a non-living state, and find that a spatially extended system with moderate diffusion can greatly facilitate the emergence of the living state. Most importantly, in this regime, the probability of emergence scales with the size of the system, so that the emergence of life somewhere is virtually guaranteed for sufficiently large systems. Overall, this is a very nice contribution. The modeling is sound, and the authors use multiple avenues (different models, different modeling approaches) to support their theory.

In the expression *k = k*_*0*_*f*, wouldn’t *f* be vanishingly small for standard RNA sequences, possibly so small that even a large *k*_*0*_ would not be sufficient to yield a reasonable *k*? The authors give some qualitative discussion of this topic towards the end of their paper, and I’d like to see the arguments fleshed out a bit more and possibly made quantitative. Do we have a sense of what a reasonable *k*_*0*_ would look like, and what k needs to be to make the origin of life happen within, say, a billion years? What would the corresponding *f* be, and is it reasonable?

Response: *We agree that if only a single sequence is functional and if the sequence is very long, then f would be very small. We also agree that if f is very small then k*_*0*_*must be large. However, it is probable that many sequences folding to the same secondary structure would have had similar functions, which makes f larger. This argument also suggests that it may be easier to begin with rather short ribozymes with a rather low catalytic ability than with longer, but rarer sequences that are better catalysts. These questions are clearly important, but we do not know how to put concrete numbers on these quantities. To give a quantitative answer to the time scale and required reaction rates, we would need to the concentrations of reagents on the primitive Earth, the reaction pathways by which nucleotides and RNAs were synthesized, and the temperature, pressure and pH at which these reactions were occurring. Current knowledge of these things is very incomplete.*

Comment: I think the manuscript would improve if it had a separate Methods section containing some of the more technical details of the simulations and derivations. Section 4 in particular mixes results with highly specific implementation details that don’t move the story forward. Similarly, other sections contain material that doesn’t directly impact the overall story, such as the proof that the Two’s Company model agrees with Eq. (*5*). I’m not asking the authors to delete this material, just reorganize it such that the a reader can get the most important results without also having to read all the supporting evidence and materials.

Response: *A methods section has been added at the end of the paper and the technical sections have been moved to the methods. The separate sections on the Two’s Company have now been merged into one main section.*

Reviewer 3: Nobuto Takeuchi, National Center for Biotechnology Information, National Library of Medicine, National Institutes of Health, USA. (Nominated by Eugene Koonin).

Comment: In their manuscript, Wu and Higgs present a minimal replicator model that exhibits a stochastic transition from a “non-living” to a “living” state. Such a transition was originally proposed and investigated by Dyson in a different model [32,49]. Using this minimal model, the authors investigate the factors determining the waiting time for such a transition. In particular, the authors calculate the waiting time as a function of the diffusion rate of replicators. The calculation reveals that the waiting time is smallest when the diffusion rate assumes an intermediate value. This result is intuitively explained by the authors as follows: Too strong diffusion diminishes the stochasticity of dynamics and so impedes the transition, whereas too weak diffusion prevents replicators from encountering each other and, thus, from catalyzing each other’s replication. Moreover, the authors examine the waiting time as a function of the system size. The calculation shows that the waiting time decreases as the system size increases, provided that the diffusion is finite. This result sharply contrasts with that obtained for infinite diffusion. The authors again give an intuitive explanation: If diffusion is finite, a non-living-to-living transition first occurs in a local region and then propagates through the entire system; thus, the larger the system, the greater the number of local regions (assuming finite diffusion), and so is the probability of a transition per unit time. Also, the authors check this result in a more complex model that considers RNA polymerization.

The manuscript describes its content in a clear manner. I obtained only one specific question regarding the results (more general comments to follow). The transition time is shortest when the diffusion rate takes an intermediate value in the minimal replicator model. Does this result also hold in the RNA polymerization model in general?

Response: *Yes, although we have not fully investigated the regime of very low diffusion rate in the RNA polymerization model. Very low diffusion rate should be unfavourable because the catalysts can only be effective if monomers and growing chains encounter the catalysts and if newly created catalysts can spread beyond the site in which they were created.*

Comment: I have three general comments (and a question). First, the non-living-to-living transition exhibited by the models rests on the combination of the two processes: the stochastic occurrence of rare events and the subsequent deterministic amplification thereof. Interestingly, such a combination also plays an important role for the stability of a spatially extended RNA-like replicator system [40]. Namely, the stable coexistence between catalysts and parasites is enabled by the formation of traveling wave patterns, whose emergence rests on the combination of the two processes: rare stochastic events in which a few catalysts are spatially segregated from parasites and the subsequent expansion of these catalysts through replication (followed by infection with parasites). This kind of dynamics, in which rare stochastic events are deterministically amplified, might be a generic feature of (life-like) systems that have a finite system size and positive feedback (see also the last paragraph).

Response: *We agree that it is interesting to note that stochastic effects and limited spatial diffusion favour both the origin of life and the stable coexistence of replicators and parasites.*

Comment: My second comment is concerned with the implication of the current study in relation to Dyson’s study [32,49]. Dyson’s model is conceived under the metabolism-first scenario for the origin of life: it is created as a simplest model of metabolic systems and assumes no replicators. By contrast, the minimal replicator model investigated in the current study, of course, assumes replicators. The RNA polymerization model of Wu and Higgs is conceived under the RNA world hypothesis, but does not actually assume replicators (i.e., it does not assume template-directed polymerization). Despite these differences, all these models exhibit essentially the same general result: two stable states that are regarded as non-living or living and stochastic transitions between them. Therefore, the general result seems to be independent of whether the model assumes metabolism, replicators, or the RNA world. This independence is also implied by the reason why these models produce the same result. Wu and Higgs identify two features shared by their models that are essential for the general result: resource limitation and nonlinear (positive) feedback—the latter does not necessarily imply replication. These features are clearly shared by Dyson’s model as well. Taken together, the results of the current study seems to imply that Dyson’s proposal of identifying the origin of life as a stochastic transition between two stable states is orthogonal to whether one considers the metabolism-first scenario, the replicator-first scenario, or the RNA world hypothesis.

Response: *Although we like the idea of the stochastic transition to life that was introduced first by Dyson, we feel that Dyson was not clear on what was meant by the ‘active’ and ‘inactive’ states and why the active state corresponds to life. In both our RNA polymerization model and the Two’s Company model, the high concentration of catalysts in the living state is maintained by the reactions carried out by the catalysts. The definition of the living state in our models is that it has autocatalytic biopolymers. Although we could presumably write down a metabolism-only model without biopolymers and without replication that would have two stable states and a possibility of a stochastic transition, this would be a purely chemical system and it would not constitute a transition to life, in our view, because the high-concentration state would not have replication, heredity and evolutionary potential.*

Comment: My last comment is concerned with the authors’ treatment of the linear growth term in the minimal replicator model. In investigating this model, the authors regard this term as less important than the other terms. However, I find three arguments to challenge this view. First, the linear growth term can play an important, though negative, role for the reported results of the model. Namely, if the linear growth rate r is greater than the decay rate u, the model has only one stable state (Figure 2 shows results for r < u). Second, replicators whose growth is described by a linear term (exponentially growing replicators, for short) are theoretically the simplest possible replicators [50]. Moreover, they have been experimentally synthesized [12].

The last argument concerns the very core of the current study. The non-living-to-living transition conceived by the authors entails rare stochastic events and the deterministic amplification thereof. Such a transition might be exhibited by exponentially growing replicators as well if one abandons the presupposition that the non-living state must be dynamically stable, as follows. Let us suppose that RNA molecules are randomly synthesized as assumed in the RNA polymerization model. Moreover, some RNA sequences are capable of self-replication; however, these sequences form but a tiny subset of the sequence space. Now, the non-living state corresponds to the state in which the system hardly contains any self-replicating molecules; the living state, the state in which the system contains self-replicating molecules in abundance. The rare stochastic event corresponds to the emergence of a (few) self-replicating molecule(s) through random synthesis; the deterministic amplification, the expansion of the nascent population of these molecules through self-replication. This argument suggests a topic for further discussion: Must “non-living” states be dynamically stable?

Response: *If r > u in our Two’s Company model, there will be no non-living state and there will be spontaneous multiplication of replicators without any waiting. This does not seem realistic to us. It is important to have non-linearity in some way in order to have two stable states. In the Two’s Company model there is only one way to do this, which is the quadratic term representing a two-molecule replication process. However, in more complex models there are many ways to do it. In the RNA polymerization models, the nonlinearity can arise either by feedback in the polymerization rate or in the monomer synthesis rate [*[27]*]. It is true that the ligase reaction in [*[12]*] corresponds to an exponentially growing replicator, but this system only works if it is supplied with very specific complementary oligomer strands. This example does not seem very close to the r reaction in our model. For the r reaction, we envisage a template directed process (e.g. on the surface of a clay catalyst) in which a single strand acts rather passively as a template. Although the template ability of different RNAs might depend to some extent on the sequence, it seems likely that most sequences could act as a passive template to some degree. If the mineral catalyzed r process was already good enough to support exponential growth (r > u), then there would be immediate multiplication of large numbers of RNAs with no sequence specificity, which seems unreasonable. The reviewer’s suggestion that the non-living state need not be dynamically stable makes sense as a logical possibility, but it seems unlikely to us, because the origin of life would just be too easy in this case.*

The possible interplay between template directed processes and ribozyme-catalyzed processes is interesting and is not fully captured in the models we studied so far. In our view, a non-specific *r* process could be important in generating diversity of RNA strands prior to life, but life itself would only arise when specific ribozymes came along to carry out the *k* process. Note that the ligase replicator in [12] is not acting as a template to specify the sequence of the new strand, because the sequence is already specified in the oligomer strands that are supplied to it. For an RNA strand to act as a catalyst it needs to fold to a specific structure. It is difficult to see how any strand could be a folded catalyst and an unfolded template at the same time. Therefore we are led almost inevitably to consider two-molecule replication processes.

Reply to the authors’ reply: *Given the persistent differences in the definitions of life, it is all the more interesting that these models—conceived under different views of life—produce the same general result based on the same principle. The authors consider that there is unlikely to be a replicator that grows exponentially and requires specific sequence patterns to act both as a catalyst and as a template (I assumed such replicators when I suggested that the non-living state need not be dynamically stable). The ligase replicator of Lincoln and Joyce (2009) is an example of such replicators as elaborated below. Although it does not answer the question I raised (its growth requires substrates prepared by humans), it suggests great possibilities of what RNA molecules can do (it was generated by (only) six rounds of in vitro selection).*

Contrary to the authors’ interpretation, the ligase replicator of Lincoln and Joyce (2009) does act as a template to specify the sequence of the new strand as follows. Their replicator consists of two ligases (denoted by E and E’), each composed of two substrates (denoted by A and B, and A’ and B’, respectively). The substrates A and B’ contain sequences complementary to each other, and so do the substrates A’ and B. Based on this complementarity, each ligase (say E) forms a complex with two substrates (A’ and B’) and catalyzes the ligation between them, synthesizing the other ligase (E’). In this way, a ligase acts both as a template and as a catalyst for the synthesis of the other ligase. More important, Lincoln and Joyce (2009) generated 12 sets of distinct substrates (denoted by An, Bn, A’n, and B’n where n ranges from 1 to 12). During a serial transfer experiment, base pair mismatches generate “recombinants” such as a ligase composed of A5 and B3 (denoted by E5,3). Such a recombinant can replicate; for example, an E5,3 forms a complex with A’3 and B’5 and catalyzes the synthesis of an E’3,5, which, in turn, catalyzes the synthesis of an E5,3. In this way, the replicator can transmit “genetic information”, thanks to the ability to act as a template.

## References

[B1] Joyce GF Deamer DW, Fleischaker GR Foreword in *Origins of Life* The Central Concepts Boston, MA: Jones and Bartlett

[B2] Gilbert W Origin of life - the RNA world Nature 1986 319 618

[B3] Bartel DP Unrau PJ Constructing an RNA world Trends Cell Biol 1999 9 12 M9 M13 10.1016/S0962-8924(99)01669-4 10611672

[B4] Joyce GF The antiquity of RNA-based evolution Nature 2002 418 214 221 10.1038/418214a 12110897

[B5] Orgel LE Prebiotic chemistry and the origin of the RNA world Crit Rev Biochem Mol Biol 2004 39 99 123 10.1080/10409230490460765 15217990

[B6] Unrau PJ Bartel DP RNA-catalysed nucleotide synthesis Nature 1998 395 260 263 10.1038/26193 9751052

[B7] Johnston WK Unrau PJ Lawrence MS Glasner ME Bartel DP RNA-catalyzed RNA polymerization: accurate and general RNA-templated primer extension Science 2001 292 1319 1325 10.1126/science.1060786 11358999

[B8] McGinness KE Wright MC Joyce GF Continuous in vitro evolution of a ribozyme that catalyzes three successive nucleotidyl addition reactions Chem Biol 2002 9 585 596 10.1016/S1074-5521(02)00136-9 12031665

[B9] Zaher HS Unrau PJ Selection of an improved RNA polymerase ribozyme with superior extension and fidelity RNA 2007 13 1017 1026 10.1261/rna.548807 17586759 PMC1894930

[B10] Hayden EJ von Kiedrowski G Lehman N Systems chemistry on ribozyme self-construction: evidence for anabolic autocatalysis in a recombination network Angew Chem Int Ed 2008 47 8424 8428 10.1002/anie.200802177 18780409

[B11] Vicens Q Cech TR A natural ribozyme with 3 ′,5 ′ RNA ligase activity Nat Chem Biol 2009 5 97 99 10.1038/nchembio.136 19125157 PMC2897744

[B12] Lincoln TA Joyce GF Self-sustained replication of an RNA enzyme Science 2009 323 1229 1232 10.1126/science.1167856 19131595 PMC2652413

[B13] Cheng LKL Unrau PJ Closing the circle: replicating RNA with RNA Cold Spring Harb Perspect Biol 2010 10.1101/cshperspect.a002204 PMC2944364 20554706

[B14] Wochner A Attwater J Coulson A Holliger P Ribozyme-catalyzed transcription of an active ribozyme Science 2011 332 209 212 10.1126/science.1200752 21474753

[B15] Ferris JP Hill AR Liu RH Orgel LE Synthesis of long prebiotic oligomers on mineral surfaces Nature 1996 381 59 61 10.1038/381059a0 8609988

[B16] Kawamura K Ferris JP Clay catalysis of oligonucleotide formation: kinetics of the reaction of the 5 ′-phosphorimidazolides of nucleotides with the non-basic heterocycles uracil and hypoxanthine Origins Life Evol Biosph 1999 29 563 591 10.1023/A:1006648524187 10666741

[B17] Ertem G Montmorillonite, oligonucleotides, RNA and origin of life Origins Life Evol Biosph 2004 34 549 570 10.1023/b:orig.0000043130.49790.a7 15570708

[B18] Rajamani S Vlassov A Benner S Coombs A Olasagasti F Deamer D Lipid-assisted synthesis of RNA-like polymers from mononucleotides Origins Life Evol Biosph 2008 38 57 74 10.1007/s11084-007-9113-2 18008180

[B19] Rajamani S Ichida JK Antal T Treco DA Leu K Nowak MA Szostak JW Chen IA Effect of stalling after mismatches on the error catastrophe in nonenzymatic nucleic acid replication J Am Chem Soc 2010 132 5880 5885 10.1021/ja100780p 20359213 PMC2857888

[B20] Costanzo G Pino S Ciciriello F Di Mauro E Generation of long RNA chains in water J Biol Chem 2009 284 33206 33216 10.1074/jbc.M109.041905 19801553 PMC2785163

[B21] Powner MW Gerland B Sutherland JD Synthesis of activated pyrimidine ribonucleotides in prebioticaly plausible conditions Nature 2009 459 239 242 10.1038/nature08013 19444213

[B22] Olasagasti F Kim HJ Pourmand N Deamer DW Non-enzymatic transfer of sequence information under plausible prebiotic conditions Biochimie 2011 93 556 561 10.1016/j.biochi.2010.11.012 21130835

[B23] Shapiro R Small molecule interactions were central to the origin of life Quart Rev Biol 2006 81 105 125 10.1086/506024 16776061

[B24] Segre D Lancet D Composing Life EMBO Rep 2000 1 217 222 10.1093/embo-reports/kvd063 11256602 PMC1083737

[B25] Bernhardt HS The RNA world hypothesis: the worst theory of the early evolution of life (except for all the others) Biol Direct 2012 7 23 10.1186/1745-6150-7-23 22793875 PMC3495036

[B26] Wu M Higgs PG Origin of self-replicating biopolymers: autocatalytic feedback can jump-start the RNA world J Mol Evol 2009 69 541 554 10.1007/s00239-009-9276-8 19777150

[B27] Wu M Higgs PG Comparison of the roles of nucleotide synthesis, polymerization and recombination in the origin on autocatalytic sets of RNAs Astrobiology 2011 11 895 906 10.1089/ast.2011.0679 22059642

[B28] Wu M Higgs PG Autocatalytic replication and homochirality in biopolymers: is homochirality a requirement of life or a result of it? Astrobiology 2012 in press 10.1089/ast.2012.0819 22931294

[B29] Ohtsuki H Nowak MA Prelife catalysts and replicators Proc Roy Soc B 2009 276 3783 3790 10.1098/rspb.2009.1136 PMC2817291 19692408

[B30] Manapat M Ohtsuki H Bürger R Nowak MA Originator dynamics J Theor Biol 2009 256 586 595 10.1016/j.jtbi.2008.10.006 18996397 PMC2674798

[B31] Manapat ML Chen IA Nowak MA The basic reproductive ratio of life J Theor Biol 2010 263 317 327 10.1016/j.jtbi.2009.12.020 20034501 PMC2827768

[B32] Dyson F Origins of Life 1999 Cambridge, UK: Cambridge University Press

[B33] Eigen M McCaskill J Schuster P The molecular quasispecies J Phys Chem 1988 92 6881 6891 10.1021/j100335a010

[B34] Altmeyer S McCaskill JS Error threshold for spatially resolved evolution in the quasispecies model Phys Rev Lett 2001 86 5819 5822 10.1103/PhysRevLett.86.5819 11415366

[B35] McCaskill JS Füchslin RM Altmeyer S The stochastic evolution of catalysts in spatially resolved molecular systems Biol Chem 2001 382 1343 1363 11688718 10.1515/BC.2001.167

[B36] Boerlijst MC Hogeweg P Spiral wave structure in pre-biotic evolution - hypercycles stable against parasites Physica D 1991 48 17 28 10.1016/0167-2789(91)90049-F

[B37] Boerlijst MC Hogeweg P Spatial gradients enhance persistence of hypercycles Physica D 1995 1995 88 29 39

[B38] Szabo P Scheuring I Czaran T Szathmary E In silico simulations reveal that replicators with limited dispersal evolve towards higher efficiency and fidelity Nature 2002 2002 420 340 343 12447445 10.1038/nature01187

[B39] Konnyu B Czaran T Szathmary E Prebiotic replicase evolution in a surface bound metabolic system BMC Evol Biol 2008 8 267 10.1186/1471-2148-8-267 18826645 PMC2575217

[B40] Takeuchi N Hogeweg P The role of complex formation and deleterious mutations for the stability of RNA-like replicator systems J Mol Evol 2007 65 668 686 10.1007/s00239-007-9044-6 17955153

[B41] Takeuchi N Hogeweg P Multilevel selection in models of prebiotic evolution II: a direct comparison of compartmentalization and spatial self-organization PLoS Comput Biol 2009 5 17 10.1371/journal.pcbi.1000542 PMC2757730 19834556

[B42] Takeuchi N Hogeweg P Evolutionary dynamics of RNA-like replicator systems: a bioinformatic approach to the origin of life Phys Life Rev 2012 in press 10.1016/j.plrev.2012.06.001 PMC3466355 22727399

[B43] Brogioli D Marginally stable chemical systems as precursors to life Phys Rev Lett 2010 105 058102 20867955 10.1103/PhysRevLett.105.058102

[B44] Ma WT Yu CW Zhang WT Monte Carlo simulation of early molecular evolution in the RNA world Biosystems 2007 90 28 39 10.1016/j.biosystems.2006.06.005 17014951

[B45] Ma WT Yu CW Zhang WT Hu JM A simple template-dependent Ligase Ribozyme as the RNA replicase emerging first in the RNA world Astrobiology 2010 10 437 447 10.1089/ast.2009.0385 20528198

[B46] Walker SI Grover MA Hud NV Universal sequence replication, reversible polymerization and early functional biopolymers: a model for the initiation of prebiotic sequence evolution PLoS One 2012 7 4 e34166 10.1371/journal.pone.0034166 22493682 PMC3320909

[B47] Wattis JAD Coveney PV The origin of the RNA world: a kinetic model J Phys Chem B 1999 103 4231 4250 10.1021/jp983159v

[B48] Buick R Sullivan WT, Baross JA The earliest records of life on Earth Planets and Life: The emerging science of Astrobiology 2007 Cambridge University Press

[B49] Dyson FJ A model for the origin of life J Mol Evol 1982 18 344 350 10.1007/BF01733901 7120429

[B50] Eigen M Self-organization of matter and the evolution of biomolecules Naturwissenschaften 1971 58 465 523 10.1007/BF00623322 4942363

